# Outcomes and management strategies of pregnancies after heart and lung transplantation across Europe

**DOI:** 10.1016/j.jhlto.2026.100481

**Published:** 2026-01-07

**Authors:** Jildau R. Meinderts, Linda W. van Laake, Dieuwertje Ruigrok, Olivier Manintveld, Jérôme Le Pavec, Michael Perch, Jens Gottlieb, Adelheid Görler, Robin Vos, Jesper M. Magnusson, Claire Merveilleux du Vignaux, Aniko Bohács, Balazs Sax, Tanel Laisaar, Vincent Bunel, Margriet E. Gosselink, Merel E. Hellemons, Kevin Damman, Jelmer R. Prins, A. Titia Lely, Erik A.M. Verschuuren, Margriet F.C. de Jong

**Affiliations:** a Department of Nephrology, Groningen Institute for Organ Transplantation, University of Groningen, University Medical Center Groningen, Groningen, The Netherlands; b Department of Cardiology, University Medical Center Utrecht and Utrecht University, Utrecht, The Netherlands; c Department of Pulmonology, University Medical Center Utrecht and Utrecht University, Utrecht, The Netherlands; d Department of Cardiology, Erasmus MC, Cardiovascular Institute, Thorax Center, Rotterdam, The Netherlands; e Erasmus MC Transplant Institute, Erasmus MC, University Medical Center Rotterdam, Rotterdam, The Netherlands; f Department of Pulmonology, Marie Lannelongue Hospital, Le Plessis Robinson, France; g Department of Cardiology Section for Lung Transplantation, Heart Center, Rigshospitalet, Copenhagen, Denmark; h Department of Clinical Medicine, University of Copenhagen, Copenhagen, Denmark; i Department of Respiratory Medicine, Hannover Medical School, Member of the German Center for Lung Research, Hannover, Germany; j Department of Cardiothoracic, Transplantation and Vascular Surgery, Hannover Medical School, Hannover, Germany; k Department of Respiratory Diseases, Department of CHROMETA, BREATHE, KU Leuven, University Hospitals Leuven, Leuven, Belgium; l Department of Respiratory Medicine, Institute of Medicine, Sahlgrenska Academy, University of Gothenburg, Gothenburg, Sweden; m Department of Respiratory Medicine, Hospices Civils de Lyon, Louis-Pradel Hospital, Lyon, France; n Department of Pulmonology, Semmelweis University, 1083 Budapest, Hungary; o Heart and Vascular Center, Semmelweis University, Budapest, Hungary; p Lung Clinic, Tartu University Hospital, Puusepa 8, 50406 Tartu, Estonia; q APHP.Nord-Université de Paris, Hôpital Bichat-Claude Bernard, Service de Pneumologie B et Transplantation Pulmonaire, Paris, France; r Department of Obstetrics and Gynaecology, University Medical Center Utrecht, Utrecht University, Utrecht, The Netherlands; s Department of Respiratory Medicine, Erasmus MC University Medical Center, Rotterdam, The Netherlands; t Department of Cardiology, University Medical Centre Groningen, University of Groningen, Groningen, The Netherlands; u Department of Obstetrics and Gynaecology, University Medical Center Groningen, University of Groningen, Groningen, The Netherlands; v Department of Pulmonary Diseases and Tuberculosis, University Medical Center Groningen, University of Groningen, Groningen, The Netherlands

**Keywords:** Post-transplantation pregnancy, Pregnancy outcomes, Long-term outcomes, Heart transplantation, Lung transplantation, Management

## Abstract

**Background:**

Literature on pregnancy after heart and/or lung transplantation (heart transplantation (HTx), lung transplantation (LTx), heart lung transplantation (HLTx)) remains sparse. This study assessed short- and long-term outcomes of pregnancies post-thoracic organ transplantation across Europe and analyzed center management of these patients.

**Methods:**

European centers provided retrospective data on post-HTx/LTx pregnancies and center strategies for handling transplant recipients with a pregnancy wish. Descriptive statistics and linear regression analysis were used.

**Results:**

Forty-two females had 50 pregnancies across 12 European centers. Pregnancy-induced hypertension occurred in 50% (HTx), 28% (LTx), and 20% (HLTx), and preeclampsia in 19%, 16%, and 20%, respectively. Preterm birth (<37 weeks) occurred in 23% (HTx) and 68% (LTx), and birth weight <2,500 g in 8% and 58%, respectively. Live birth rate was 98%. In multivariable analysis, a trend for higher birth weight with higher pre-pregnancy estimated glomerular filtration rate was observed (B 13.3, 95% CI −1.7-28.4, *p* = 0.08). Graft function remained stable in most patients during and after pregnancy. During follow-up (mean 15 years, range 5-31 post-transplantation), 6/40 mothers (15%) died (1 HTx, 2 LTx, 3 HLTx), with their children aged 0-11 years. No specific physical health problems were mentioned in 29/30 children (age 0-22 years). Physician opinions towards pregnancy differed from reluctant (31%) to positive (69%), with numerous management variations.

**Conclusions:**

We show reassuring pregnancy outcomes for post-HTx, LTx, and HLTx patients in an exclusive European cohort, despite high pregnancy complication rates. Graft function and overall maternal survival appear unaffected. We highlight differences in pregnancy management between centers and suggest development of a uniform approach.

## Background

Almost all current knowledge on pregnancy after transplantation originates from kidney or liver transplantation.^1^, ^2^, ^3^ Data about pregnancy after thoracic organ transplantation remains scarce. Most data comes from the Transplant Pregnancy Registry International (TPRI), a voluntary registry with mostly American data.^1^ Data from European patients is limited to small case series and cohort studies.^1^, ^4^, ^5^ To optimally guide patients after heart, lung, or combined heart-lung transplantation (HTx, LTx, and HLTx, respectively) with a pregnancy wish, it is important to understand both short-term and long-term risks.

On the short-term pregnancy after thoracic organ transplantation carries general risks of a pregnancy after transplantation, including an increased risk of hypertensive disorders of pregnancy (including preeclampsia), preterm birth, and low birth weight. Some studies report similar risks to pregnancies after kidney and liver transplantation, and some report significantly higher risks.^4^, ^5^ Thoracic grafts are directly affected by hemodynamic changes of pregnancy, which may increase the risk of pregnancy complications and graft dysfunction. Besides, regardless of the pregnancy, overall survival after HTx and certainly after LTx is much shorter than after kidney or liver transplantation, impacting long-term family outcomes. This indicates that pregnancies after thoracic organ transplantation might need a different approach and counseling.

The primary aim of this study was to assess both short- and long-term outcomes of pregnancies after HTx and/or LTx across Europe. Furthermore, we explored how centers across Europe approach a transplant recipients with a pregnancy wish after HTx, LTx, or HLTx with 3 general management questions.

## Methods

### Study design

In this international multicenter cohort study, European HTx and/or LTx centers were invited to participate via email (sent to 85 physicians) and at the European Society for Heart and Lung Transplantation congress in 2024. The study had a 2-staged approach. First, centers answered 3 general questions about their management of pregnancy after HTx, LTx, or HLTx (Supplemental Table 1). Secondly, centers submitted anonymized pregnancy data (>20 weeks of gestation) in an online database. Centers without pregnancies only completed the first phase. This cohort reflects a convenience sample of centers across Europe that were able to participate. As pregnancy after thoracic transplantation is rare, cases ≥20 weeks of gestation were included from any contributing center, irrespective of program size. The study was approved by the local ethical review board of the University Medical Center Groningen (METc-2023/294), and additional informed consent was obtained in participating centers if applicable. All procedures were conducted in accordance with the declarations of Helsinki and Istanbul.

### Data collection

Study data were collected and managed using REDCap electronic data capture tools hosted at the University Medical Center Groningen.^6^, ^7^ Data included demographics, details of transplantation, pre-pregnancy comorbidities, pregnancy complications, and health status at last follow-up for mother and child according to the transplant physician. Estimated glomerular filtration rate (eGFR) was calculated using CKD-EPI 2009.^8^ To assess the impact of pregnancy on graft function, yearly data on HTx/LTx parameters were collected from 3 years pre-conception to 3 years post-delivery.

### Definitions

Complications were included when mentioned as such by the physician.

Furthermore, the following definitions were used:

Preterm delivery: delivery <37 weeks of gestation.^9^

Low birth weight (LBW): birth weight <2,500 gram.^10^

− Very LBW: birth weight <1,500 gram.^10^
− Small for gestational age: birth weight <10th percentile. Percentiles according to the World Health Organization fetal growth calendar.^11^

### Statistical analyses

Due to the rarity of pregnancies after thoracic transplantation, HTx, LTx, and HLTx cases were analyzed together. However, all outcomes for HTx and LTx recipients are presented separately throughout the manuscript. HLTx cases were too few for subgroup analysis and are reported descriptively. Statistical analyses were performed with SPSS version 28.0.1.0 (IBM, Armonk, NY). Descriptive statistics were used to characterize the study population. Normality of distribution was tested with the Shapiro-Wilk test. Continuous variables are presented as mean (standard deviation (SD)), median [interquartile range (IQR)] where applicable, and categorical variables as frequencies with percentages.

Linear regression analysis was performed to evaluate the association between pre-pregnancy characteristics (pre-pregnancy eGFR, BMI, and transplantation type) and pregnancy outcomes (birth weight and gestational age). Variables with a *p*-value <0.20 in univariable analysis were included in a multivariable linear regression model.

## Results

### Population and demographics

In total 12 centers from 8 countries participated (Figure S1). Two centers did not report a pregnancy and only answered the general questions. In total we included 50 pregnancies between 1999 and 2024 in 42 patients (Table 1). The number of births increased over time (Figure S1). In the HTx group cardiomyopathy was the most common reason for transplantation (58%), followed by congenital heart disease (17%), and in the LTx group infectious causes, including cystic fibrosis (58%), followed by vascular causes (38%). All patients with a HLTx had a congenital cause for their transplantation. The median age at transplantation was 24 years for HTx and LTx and 22 for HLTx. Mean eGFR before first pregnancy was 90 for the HTx, 80 for the LTx and 91 ml/min/1.73 m^2^ for the HLTx group. Eight patients (2 HTx, 6 LTx) had an eGFR <60 ml/min/1.73 m^2^ pre-pregnancy. Pre-pregnancy BMI was <18.5 kg/m^2^ in 7 patients (1 HTx, 5 LTx, and 1 HLTx) and >30 kg/m^2^ in 5 (3 HTx, 2 LTx).

**Table 1:** Demographics and Clinical Characteristics

			HTx	LTx	HLTx
*Patients*			12	24	6
Pregnancies after transplantation			16	27	7
Indication for LTx	Infectious		NA	14/24 (58)	0/5 (0)
	Fibrosis		NA	1/24 (4)	0/5 (0)
	Vascular		NA	9/24 (38)	0/5 (0)
	Variable		NA	0/24 (0)	5/5 (100)
Indication for HTx	Cardiomyopathy		7/12 (58)	NA	0/5 (0)
	Congenital heart disease		2/12 (17)	NA	5/5 (100)
	Myocarditis		1/12 (8)	NA	0/5 (0)
	Other		2/12 (17)	NA	0/5 (0)
*Year of transplantation*			2006 (7)	2010 (6)	2004 (7)
Age at transplantation (years)			24 (4)	24 (4)	22 (4)
	<18 years of age		4/12 (33)	2/24 (8)	0/6 (0)
Comorbidities before pregnancy	Hypertension		1/12 (8)	3/24 (13)	0/5 (0)
	Diabetes Mellitus		1/12 (8)	7/24 (29)	0/5 (0)
	eGFR (ml/min/1.73 m^2^)		90 (23)	80 (21)	91 (93)
		eGFR <60 ml/min/1.73 m^2^	2/11 (18)	6/22 (27)	0/5 (0)
BMI before first pregnancy (kg/m^2^)			24.7 (5)	22.3 (5)	22.0 (5)
	<18.5 (kg/m^2^)		1/12 (8)	5/23 (22)	1/4 (25)
	>30 (kg/m^2^)		3/12 (25)	2/23 (9)	0/4 (0)

Data described as number/total number (%) or mean (standard deviation) were applicable. Definitions: eGFR: CKD-EPI 2009 formula used. Abbreviations: BMI, body mass index; HLTx, heart lung transplantation; HTx, heart transplantation; LTx, lung transplantation; NA, not applicable.

### Maternal pregnancy outcomes

Table 2 describes characteristics per pregnancy. In total, 16 pregnancies after HTx, 27 after LTx and 7 after HLTx were included. In most pregnancies (*n* = 34, 68%), preconception counseling was performed, but in 14% after HTx and in 24% after LTx, the pregnancy was unplanned from the doctor’s perspective. Pregnancy-induced hypertension occurred in 50%, 28%, and 20% and preeclampsia in 19%, 16%, and 20% in HTx, LTx and HLTx, respectively. Of the 7 women (8 pregnancies) with preeclampsia, 4 had a pre-pregnancy eGFR <60 ml/min/1.73 m^2^ and the other 3 an eGFR of 61, 63, and 72 ml/min/1.73 m^2^. One pregnancy was complicated by Hemolysis Elevated Liver enzymes and Low Platelets (HELLP) syndrome and 1 pregnancy by eclampsia*.* No graft rejection or other graft problems during pregnancy were reported. One patient developed post-transplant lymphoproliferative disease during pregnancy. She delivered her child at 27 weeks of gestation with very LBW and died within 1 year after delivery due to therapy-resistant progression of post-transplant lymphoproliferative disease.

**Table 2:** Pregnancy Outcomes Per Pregnancy

			HTx	LTx	HLTx
Total pregnancies post-transplantation			16	27	7
	First pregnancy		12	24	6
	Second pregnancy		4	3	1
Maternal age at conception (years)			29 (3)	30 (4)	28 (2)
Interval transplantation-conception (years)			10 [4-12]	5 [3-7]	7 [4-9]
Year of birth offspring			2018 [2013-2020]	2019 [2015-2021]	2009 [2003-2016]
Planned pregnancy	No		2/15 (14)	6/25 (24)	0/4 (0)
	Yes, without preconception counseling		0/15 (0)	0/25 (0)	2/4 (50)
	Yes, with preconception counseling		13/15 (87)	19/25 (76)	2/4 (50)
Conception	Spontaneous		15/15 (100)	20/26 (77)	4/4 (100)
	With fertility treatment		0/15 (0)	6/26 (23)	0/4 (0)
Pregnancy related complications	Hypertension		8/16 (50)	7/25 (28)	1/5 (20)
	Preeclampsia		3/16 (19)	4/25 (16)	1/5 (20)
	Gestational diabetes		2/16 (13)	2/25 (8)	0/5 (0)
	Graft rejection		0/16 (0)	0/25 (0)	0/5 (0)
Method of delivery	Vaginal		10/15 (67)	14/24 (58)	4/6 (67)
	C-section		5/15 (33)	10/24 (42)	2/6 (33)
Pregnancy outcome	Live birth		15/15 (100)	26/27 (96)	6/6 (100)
	Medical termination of pregnancy		0/15 (0)	1/27 (4)	0/6 (0)
	Neonatal death >7 days		0/15 (0)	1/27 (4)	0/6 (0)
Gestational age (weeks)*			37 [36-38]	36 [33-37]	35 (*n* = 1)
	Preterm birth <37 weeks		3/13 (23)	13/19 (68)	1/1 (100)
		<34 weeks	1/13 (8)	6/19 (32)	0/1 (0)
Birth weight (grams)*			2,889 (39)	2,420 [1,810-3,020]	2,600 and 3,400
	Low birth weight (<2,500 gs)		1/12 (8)	11/19 (58)	0/2 (0)
		Very low birth weight (<1,500 gs)	0/12 (0)	3/19 (16)	0/2 (0)
Small for gestational age (<p10)*			0/10 (0)	2/19 (11)	0/1 (0)

Short-term pregnancy outcomes during pregnancy and up to 28 days after delivery. Twin pregnancies were considered as 1 pregnancy and therefore counted once. Data given as mean (standard deviation), median [interquartile range], or number/total number (%) where appropriate.

Abbreviations: C-section, caesarean-section; HELLP, Hemolysis Elevated Liver enzymes and Low Platelets; ICSI, intracytoplasmic sperm; IUI, intrauterine insemination; IVF, in vitro fertilization; LTx, lung transplantation; HTx, heart transplantation; HLTx, heart lung transplantation; Tx, transplantation. *medical termination of pregnancy excluded.

### Fetal outcomes

Live birth rate was 98%. One termination of pregnancy for medical indication (of a twin) and 1 neonatal death (both after LTx) were reported. Excluding the termination of pregnancy, median gestational age was 37 weeks (IQR 36-38) and 36 weeks (IQR 33-37) after HTx and LTx respectively. For HLTx, gestational age was only reported for 1 child (35 weeks). In the HTx group, preterm delivery occurred in 23%, and in the LTx group 68%. Median birth weight was 2,889 g (IQR 2,565-3,307) (HTx) and 2,420 g (IQR 1,810-3,020) (LTx). LBW occurred in 8% (HTx) and in 58% (LTx). Two children (both LTx) were small for gestational age (<p10, >P3).

### Pre-pregnancy factors associated with adverse pregnancy outcome

In univariable linear regression analysis, a higher pre-pregnancy eGFR and HTx transplant type were significantly associated with a higher birth weight (B 15.2, 95% CI 0.6-29.8, *p* = 0.04 and B 470, 95% CI 26.8-912.6, *p* = 0.04, respectively) (Table 3). A similar trend, although not statistically significant was observed for gestational age (B 0.1, 95% CI −0.02-0.13, *p* = 0.12 and B 2.2, 95% CI −0.3-4.6, *p* = 0.08). No clear association was observed for BMI and gestational age or birth weight. In multivariable analysis, no significant associations were observed, although a trend for higher pre-pregnancy eGFR and higher birth weight was seen (B 13.3, 95% CI −1.7-28.4, *p* = 0.08).

**Table 3:** Linear Regression Analysis

Dependent variable	Baseline variables	B (95% CI)	*p*-value	B (95% CI)	*p*-value
		Univariable analysis		Multivariable analysis	
Birth weight (grams)	Pre-pregnancy eGFR (ml/min/1.73 m^2^)	15.2 (0.6-29.8)	**0.04**	13.3 (−1.7-28.4)	0.08
	Tx type*	470.0 (26.8-912.6)	**0.04**	301.0 (−285.8-887.8)	0.30
	BMI (kg/m^2^)	36.4 (−45.8-118.6)	0.37		
Gestational age (weeks)	Pre-pregnancy eGFR (ml/min/1.73 m^2^)	0.1 (−0.02-0.13)	0.12	0.05 (−0.03-0.12)	0.22
	Tx type*	2.2 (−0.3-4.6)	0.08	1.6 (−1.4-4.7)	0.27
	BMI (kg/m^2^)	0.2 (−0.2-0.6)	0.24		

Linear regression analysis of pre-pregnancy eGFR and BMI and Tx type (heart, lung, or combined heart lung). * Reference is lung transplantation.

Abbreviations: BMI, body mass index; CI, confidence interval; eGFR, estimated glomerular filtration rate; Tx, transplantation.

### Long-term follow-up

Mean follow-up after transplantation was 17 years (SD 7) for HTx, 13 years (SD 6) for LTx and 16 years (SD 7) for HLTx. Mean follow-up after delivery of the first child was 5 years (SD 4) for HTx, median 4 years (IQR 1-8 for LTx and mean 9 years (SD 7) for HLTx (Table 4). During follow-up 6/40 mothers (15%) died (1 HTx, 2 LTx, 3 HLTx) with their 8 children aged 0-11 years. Details of their death are depicted in Supplemental Table S2. Two patients (1 HTx, 1 LTx) were reported to have significant kidney function decline after delivery. Currently, 1 patient is in a preterminal stage of kidney insufficiency (eGFR 21 ml/min/1.73 m^2^) and 1 on dialysis. Both patients had an impaired kidney function (eGFR <60 ml/min/1.73 m^2^) pre-pregnancy and their pregnancies were complicated by preeclampsia. At the last follow-up no specific physical health problems were mentioned in 29/30 alive children (age range 0-22 years). One child had bronchopulmonary dysplasia related to very preterm birth.

**Table 4:** Long-Term Follow Up

	HTx	LTx	HLTx
Follow-up after transplantation (years)	17 (7)	13 (5)	15 (5)
Follow-up after delivery of the first child (years)	6 (4)	4 [1-8]	8 (6)
Mother deceased (yes)	1/12 (8)	2/24 (8)	3/6 (50)
Re-transplantation (yes)	0/12 (0)	2/24 (8)	0/6 (0)

Data described as number/total number (%), mean (standard deviation), or median [IQR] were applicable.

Abbreviations: HLTx, heart lung transplantation; HTx, heart transplantation; LTx, lung transplantation.

### Long-term graft function

**Figure 1:**
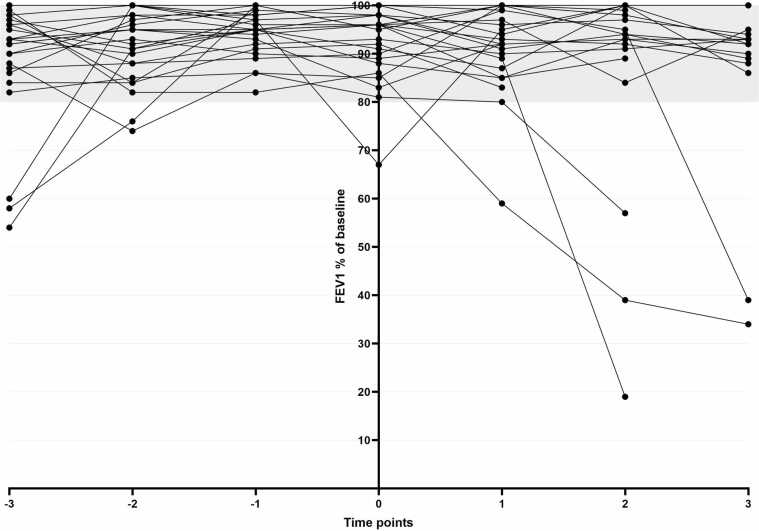
FEV1% of baseline before and after pregnancy in LTx and HLTx patients. FEV1% of baseline from −3 years before conception till 3 years after delivery of the child, time point 0 is the first measurement after delivery of the child. Number of patients is 26. Abbreviations: HLTx: heart lung transplantation, LTx: lung transplantation.

For HTx patients, including HLTx, echocardiogram parameters from 3 years before conception till 3 years after delivery of the first child after transplantation are depicted in Supplemental Table S3, with no substantial difference when comparing values before and after delivery. Rejection in the HTx group was reported only in the previously mentioned deceased HLTX patient. For LTx patients, including HLTx, in Figure 1 the FEV1% of baseline is depicted from 3 years before conception until 3 years after delivery. In almost all patients, lung function remained stable after delivery of their first child after LTx and >80% at all follow-up moments. One patient, with a complicated pregnancy with HELLP syndrome, had a drop in FEV1% shortly after delivery, but this recovered afterwards (from 97% to 67% to 95%). Chronic lung allograft dysfunction was reported in 3 LTx patients and 1 HLTx patient after delivery. In one of these patients, bronchiolitis obliterans syndrome led to successful re-transplantation 13 year after LTx and 5 years after delivery, and in 1 patient, this led to death 13 years after HLTX and 8 years after delivery (Supplemental Table S2).

### Approach and management of centers

**Figure 2:**
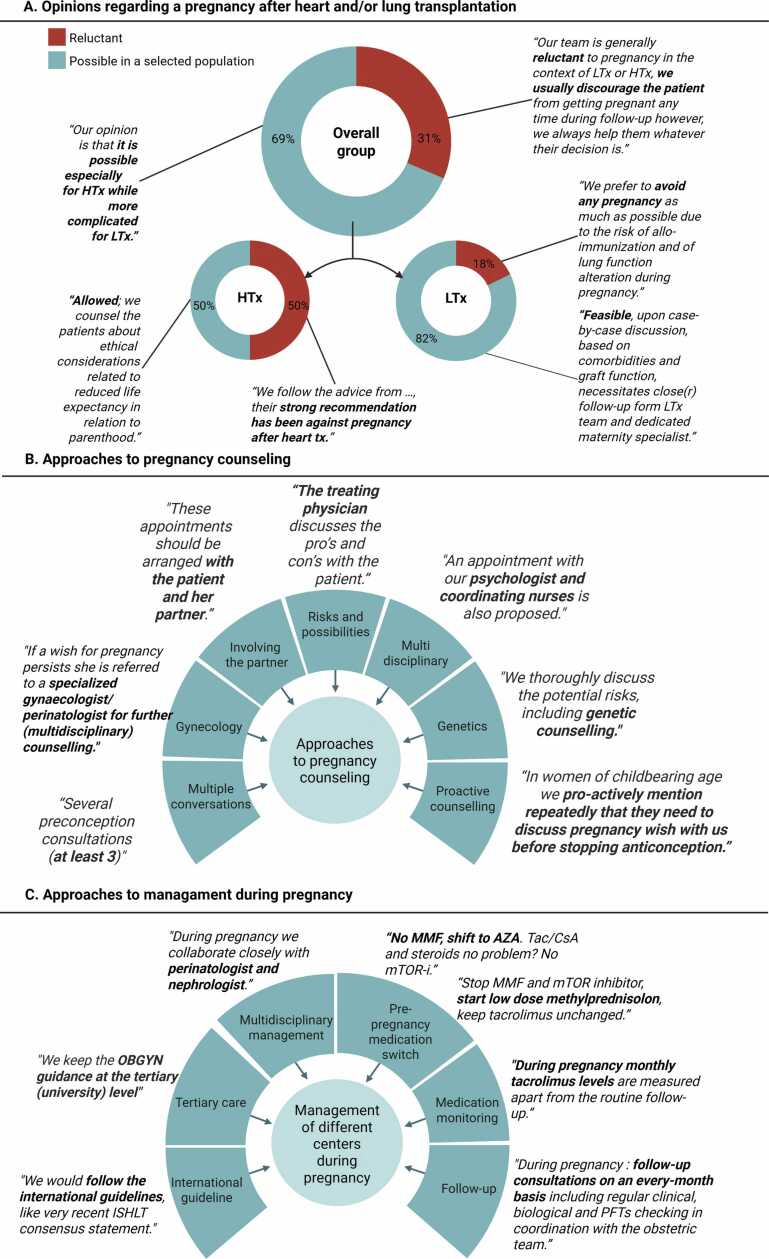
Physicians answers to questions regarding the management of their center.

In Figure 2, responses to the questions regarding center-specific management are summarized. Sixteen physicians participated, of which 4 were involved solely in HTx care, 8 in LTx care, and 4 in both HTx and LTx. Overall, distinct differences in opinions and management were observed among physicians. For question 1 (opinions on pregnancy post-transplantation), 31% (*n* = 5) of physicians were reluctant or discouraged pregnancies, with notable differences between the HTx (50% reluctant) and LTx (18% reluctant) groups (Figure 2A).

Regarding counseling of patients with a pregnancy wish (question 2, Figure 2B), most physicians emphasized counseling should be performed to discuss risks for mother and child. Multiple physicians highlighted the importance of involving the partner, and some physicians specifically mentioned that longevity after transplantation in relation to raising a child should be addressed. Approaches varied: some preferred counseling by the treating physician, others referred patients to specialized gynecologists or multidisciplinary teams, including social workers, psychologists, and coordinating nurses. Genetic counseling was frequently mentioned. One center specified that at least 3 preconception counseling sessions should be performed, and another conducted biopsies to rule out subclinical rejection in HTx patients. Two centers mentioned they proactively counseled their patients about pregnancy post-transplantation. All centers emphasized the need for close follow-up during pregnancy. One HTx center indicated the gynecologist should take the lead during pregnancy, and 1 LTx center involved a nephrologist in the multidisciplinary team.

For immunosuppressive medication (question 3, Figure 2C), centers agreed mycophenolate mofetil (MMF) should be discontinued prior to conception, with differing intervals ranging from 6 weeks to 6 months. Approaches varied, 9 physicians switched MMF to azathioprine, 2 stopped MMF, 1 switched MMF to prednisone, and 1 physician stopped MMF in case of triple therapy and switched MMF to prednisone in case of double therapy. Most centers agreed mTOR inhibitors should be discontinued, while 1 center (without pregnancies) mentioned they would replace MMF with everolimus (mTOR inhibitor).

## Discussion

This retrospective multicenter cohort study evaluated short- and long-term outcomes of pregnancies after HTx, LTx, and HLTx across Europe. In the short-term, live birth rate was high, despite an increased risk of pregnancy-related complications for both mother and fetus compared to the general population. Adverse fetal outcomes were more pronounced in the LTx group. No graft-associated pathology during pregnancy was reported. In the long-term, pregnancy does not appear to affect graft function and survival rates negatively. Patients with declined kidney function before pregnancy might be at an increased risk for both pregnancy complications and kidney function decline after delivery. A key strength of this study is the combination of clinically verified maternal and fetal outcomes with long-term follow-up and center-level management strategies, including physician perspectives on counseling and immunosuppression adjustments. This approach provides a comprehensive overview of both outcomes and real-world practice across Europe. Our findings are consistent with previously reported outcomes, which predominantly originate from the TPRI cohort with mainly American data.^1^ This suggests that pregnancy outcomes after thoracic transplantation are applicable across different healthcare systems, which is in line with global data from pregnancies after kidney transplantation.^12^

Our short-term outcomes are in line with existing literature, although slightly better results were observed in our HTx group. Preterm delivery rates in the general European population rates are estimated at 6.2%.^13^ The TPRI reports preterm birth rates of 43% in HTx recipients and 64% in LTx recipients^1^ and a recent systematic review reported 45% and 58%,^5^ compared to 23% and 68% in our cohort, respectively. LBW rates reported in the TPRI are 37% for HTx and 60% for LTx, compared to 8% and 58% in our cohort and between 5% and 8% in the general European population, depending on the region.^14^ The cause of the substantially higher rate of fetal pregnancy complications in LTx compared to HTx could be associated to the difference in pre-pregnancy kidney function, as suggested by our linear regression analysis. This is in line with existing literature on pregnancies in CKD patients,^15^ as well as studies in kidney and liver transplantation recipients, where the risk of adverse pregnancy outcome was primarily dependent on pre-pregnancy kidney function.^16^, ^17^ In contrast to previous studies, we observed no episodes of acute graft rejection during pregnancy, which may reflect the lower percentage of unplanned pregnancies in our cohort (14% HTx, 24% LTx) compared to the 38%-82% reported in earlier studies.^5^, ^18^, ^19^ Nonetheless, the number of unplanned pregnancies is still high, emphasizing the importance of frequent, pro-active attention towards to topic of reproduction.

Data on long-term outcomes are scarce, but consistent with this study, pregnancy does not appear to increase the risk of rejection, graft loss, or death in most patients.^5^ However, pregnancy itself remains high risk with complications that may impact maternal health. The long-term outcomes align with studies in kidney and liver transplant recipients, where pregnancy did not negatively affect graft function or survival.^20^, ^21^ However, making a fair comparison with non-pregnant women is challenging, as women who become pregnant after HTx or LTx represent a selected population that is generally healthy and doing well post-transplantation.^4^ Recognizing that this population likely has better survival chances might be reassuring for couples with a pregnancy wish after transplantation. Nonetheless, the median time to death/re-transplantation in HTx recipients aged 18-39 years is 13.2 years and for LTx recipients 7.0 years, with survival being slightly better in Europe compared to the overall group.^22^ This means that, even with above average survival, the majority of children will still grow up without their mother, which remains an important topic that should be discussed in the preconception counseling. Intriguingly, the likelihood of early parental death applies equally to fathers of children born after transplantation, yet this aspect tends to receive less attention.

For both HTx and LTx recipients, we show that graft function was unaffected by pregnancy in most patients, which aligns with most of the previous research. A Canadian study of 8 HTx patients with 18 pregnancies found no impact on graft function or long-term survival,^19^ and lung function remained stable in LTx patients post-pregnancy in 2 recent studies from Israel and the United Kingdom.^23^, ^24^ However, a French study in LTx patients reported that 38% (13/34 patients) had an absolute FEV1 decline of >5% at 12 months post-pregnancy.^25^ We observed significant kidney function decline in 2 women post-pregnancy, consistent with other studies in thoracic organ recipients (median follow-up between 1 and 7 years), which reported up to 1 in 4 patients with kidney function deterioration during or after pregnancy.^4^, ^19^, ^26^ In our study, this decline was seen in patients with pre-existing reduced kidney function, and both pregnancies were complicated by preeclampsia. This suggests that the risk factor may be the pre-existing kidney function rather than the transplantation itself.

Our secondary aim was to evaluate pregnancy management across European centers. We observed significant variations in opinions and practices, which may impact outcomes. These differences were as well seen in 2 questionnaire studies on HTx and LTx care with both over 100 participants. An American study reported that only 43% (*n* = 52) of centers had a formal policy regarding pregnancy following HTx.^27^ And an international survey study with 103 responses reported that 70% did not have a formal policy regarding pregnancy after maternal LTx.^28^ Previous research on pregnancies in patients with chronic diseases, including kidney transplantation, showed that patients are aware of the conflicting information provided by different centers and physicians, which contributes to increased stress.^29^, ^30^, ^31^ This underscores the importance of adopting a uniform approach when guiding patients with a pregnancy wish to ensure optimal support. Recently, the ISHLT consensus statement on pregnancy after thoracic organ transplantation was published, providing a comprehensive overview of patient management.^32^ The observed heterogeneity in counseling, medication adjustments, and multidisciplinary involvement in our study should be interpreted as an important real-world finding. These data reflect clinical practice before or during early implementation of the 2023 ISHLT consensus statement on reproductive health after thoracic transplantation and underline the need for greater standardization of care. We believe adherences to these guidelines would improve patient care across centers. Moreover, given the low numbers of pregnancies within a center, care might be further improved by centralizing management of all transplant patients with a pregnancy wish within a single specialized team, combining expertise from different solid organ transplantation types. We argue that recent studies, including our own, provide enough reassurance to reconsider the strict contraindication against pregnancy after HTx and LTx and instead assess the possibilities on an individual patient basis, considering that mothers may die at young age of their child(ren).

Our data reflect that only a minority of patients become pregnant after HTx or LTx.^19^, ^24^, ^33^ For LTx, this number may decrease further with the introduction of new medication for patients with CF and PAH.^34^, ^35^ In contrast, for HTx, numbers may increase as more pediatric HTx patients reach their reproductive years. This study underscores the value of collaboration in rare conditions like pregnancies post-HTx and LTx.

We acknowledge several limitations. Although this study includes data from 12 centers across 8 countries, the cohort represents a convenience sample. Not all major European thoracic transplant programs participated, despite efforts to broaden participation. Given the rarity of pregnancy after HTx or LTx, the overall number of cases is inevitably small, which limits statistical power and the precision of subgroup analyses, especially for HLTx. In addition, clinical practice varied between centers, which complicates direct comparison of outcomes. However, capturing this heterogeneity was a predefined aim, as little is known about real-world management across Europe. Definitions of pregnancy complications could not always be uniformly verified, and the qualitative information on management strategies reflects exploratory, self-reported data rather than systematically collected observations. Despite these limitations, this study provides, to our knowledge, the largest clinically verified European datasets to date. Important future research aims are the long-term health outcomes of the offspring born after transplantation^36^, ^37^ and the experiences of families with a pregnancy after HTx and LTx, to see if additional support is needed as is suggested in pregnancy after liver transplantation.^38^

## Conclusion

To conclude, this European cohort study demonstrates reassuring outcomes for pregnancies after HTx and/or LTx in a selected population. While pregnancy complication rates are high, almost all children are born healthy. Long-term graft and overall survival appear unaffected in most patients. Impaired pre-pregnancy kidney function might be a risk factor for adverse outcomes. Lastly, this study highlights substantial variation in the management of patients with a pregnancy wish post-HTx and/or LTx. We recommend frequent, proactive attention towards to topic of reproduction and thorough pre-pregnancy counseling by an expertise team in selected candidates based on uniform pregnancy guidelines. This makes pregnancy achievable for well-suited patients, taking into account that a considerable number of children will lose their mother early in life given the overall survival rates after HTx and LTx.

## Financial support

J.R. Meinderts, D. Ruigrok, J. le Pavec, J. Gottlieb, A. Görler, C. Merveilleux du Vignaux, A. Bohács, B. Sac, T. Laisaar, V. Bunel, M.E. Gosselink, M.E. Hellemons, J.R. Prins, A.T. Lely, E.A.M. Verschuuren, and M.F.C. de Jong have nothing to disclose. L.W. van Laake reports research grants and speaker fees to her employer by Medtronic, Abbott, Novartis, and Vifor. O. Manintveld reports speaker engagement or advisory board fees from Astra Zeneca, Abbott, Boehringer Ingelheim, Cablon Medical, Daiichi Sankyo, Novartis, Novo Nordisk, and Siemens, all unrelated to this manuscript. M. Perch has received unrestricted research grants from the PulmonX and Therakos and reports advisory board activity for Ryme Medical, Takeda, TFF and Zambon, all unrelated to this manuscript. R. Vos is supported as Senior Clinical Researcher by the Research Foundation-Flanders (1803521N); otherwise, I have no other related financial disclosures. J.M. Magnusson has received an unrestricted research grant from Boehringer Ingelheim and consulting fees from Boehringer Ingelheim, AstraZeneca, GlaxoSmithKline, Takeda Pharma, Vicore Pharma, and Mallinckrodt. Kevin Damman reports speaker and consultancy fees to his employer by AstraZeneca, Boehringer Ingelheim, Abbott, FIRE1, EchoSense, and Novartis. This research did not receive any specific grant from funding agencies in the public, commercial, or not-for-profit sectors.

## Conflicts of Interest statement

The authors declare the following financial interests/personal relationships, which may be considered as potential competing interests. L.W. van Laake reports a relationship with Medtronic, Abbott, Novartis, and Vifor that includes: funding grants and speaking and lecture fees. O. Manintveld reports a relationship with Astra Zeneca, Abbott, Boehringer Ingelheim, Cablon Medical, Daiichi Sankyo, Novartis, Novo Nordisk, and Siemens, all unrelated to this manuscript. that includes: consulting or advisory and speaking and lecture fees. R. Vos reports a relationship with Research Foundation-Flanders (1803521N) that includes: funding grants. J.M. Magnusson reports a relationship with Boehringer Ingelheim, AstraZeneca, GlaxoSmithKline, Takeda Pharma, Vicore Pharma, and Mallinckrodt that includes: consulting or advisory and funding grants. M. Perch reports a relationship with PulmonX, Therakos, Ryme Medical, Takeda, TFF, and Zambon, all unrelated to this manuscript, that includes: consulting or advisory and funding grants. K. Damman reports a relationship with AstraZeneca, Boehringer Ingelheim, Abbott, FIRE1, EchoSense, Novartis. that includes: consulting or advisory. If there are other authors, they declare that they have no known competing financial interests or personal relationships that could have appeared to influence the work reported in this paper.
