# Supplementary material for: Outcomes and management strategies of pregnancies after heart and lung transplantation across Europe
Source: JHLT Open. 2026 Jan 7;11:100481. doi: 10.1016/j.jhlto.2026.100481 (PMC12874786; doi:10.1016/j.jhlto.2026.100481)
Supplement: Supplementary file 1 — Supplementary material [file mmc1.docx]

Supplemental material

**Supplemental Table S1. General question asked to participating centers**

| Question 1: What is the opinion regarding pregnancy after heart and/or lung transplantation in your center? |
| --- |
| Question 2: How does your center approach the counseling/guidance of a pregnancy after heart and/or lung transplantation? (before and during pregnancy) |
| Question 3: What is de management regarding the immunosuppressive medication around a pregnancy after heart and/or lung transplantation in your center? |
| Question 4: Number of women and number of pregnancies after HTx: |
| Question 5: Number of women and number of pregnancies after LTx: |

**Supplemental Table S2. Characteristics of deceased patients**

Details of deceased patients *Abbreviations: HLTx: heart lung transplantation, HTx: heart transplantation, LTx: lung transplantation, Tx: transplantation*

| No | Transplantation type | Pregnancies post-Tx | Year of death | Interval Tx-death (years) | Interval delivery-death (years) | Age mother at death  (years) | Cause of death |
| --- | --- | --- | --- | --- | --- | --- | --- |
| 1 | HTx | 1 | 2013 | 15 | 11 | 37 | Unknown |
| 2 | LTx | 1 | 2022 | 12 | 0 | 34 | progressive therapy-refractory PTLD |
| 3 | LTx | 2 | 2003 | 9 | 4 & 2 | 40 | PTLD |
| 4 | Combined HLTx | 2 | 2011 | 18 | 10 & 8 | 36 | Unknown |
| 5 | Combined HLTX | 1 | 2017 | 13 | 8 | 40 | Non adherence to medication leading to CLAD |
| 6 | Combined HLTX | 1 | 2016 | 8 | 1 | 29 | Cardiac AMR with DSA targetting exclusively the heart |

**Supplemental Table S3.** **Echocardiographic parameters before and after pregnancy in HTx patients**

Echocardiographic parameters from -3 years before conception till 3 years after delivery of the child, time point 0 is the first measurement after delivery of the child. *Abbreviations: CAV: cardiac allograft vasculopathy, LVEF: left ventricular ejection fraction, NA: not available,*

|  | 3 yrs before delivery | 2 yrs before delivery | 1 year before delivery | First after delivery | 1 year after delivery | 2 yrs after delivery | 3 yrs after delivery |
| --- | --- | --- | --- | --- | --- | --- | --- |
| LVEF | Reasonable | Good | Good | Good | Good | Good | Good |
|  | Normal | Normal | >60 | >60 | >60 | >60 | >60 |
|  | Good | Good | 59% | 55% | 55% | Good | Good |
|  | 70% | 70% | 70% | 65% | 65% | 65% | 65% |
|  | 65% | 65% | 65% | 65% | NA | NA | NA |
|  | 60% | 60% | 60% | NA | NA | 60% | 50% |
|  | 60% | NA | 60% | 60% | NA | NA | 60% |
|  | 60% | 65% | 65% | 60% | 68% | NA | NA |
|  | 57% | NA | 57% | 57% | NA | NA | 60% |
|  | 55% | 55% | 55% | 60% | 55% | NA | 55% |
|  | 55% | 60% | NA | 55% | 55% | 65% | NA |
|  | NA | NA | Normal | Normal | Normal | NA | NA |
| CAV (grades 0-3) | 0 | 0 | 0 | 0 | 0 | 0 | 0 |
|  | 0 | 0 | 0 | 0 | 0 | NA | NA |
|  | 0 | 0 | 0 | 0 | 0 | 2 | 2 |
|  | 0 | 0 | 0 | 0 | NA | NA | NA |
|  | 0 | 0 | 0 | NA | NA | 0 | 0 |
|  | 0 | NA | 0 | 0 | NA | NA | 0 |
|  | 0 | 0 | 0 | 0 | NA | NA | 0 |
|  | 0 | NA | 0 | 0 | NA | NA | 0 |
|  | 0 | 0 | 0 | 0 | 0 | NA | 0 |
|  | 0 | 0 | NA | 0 | 0 | 0 | NA |

**Figure S1. Number of included pregnancies per center and per time period**


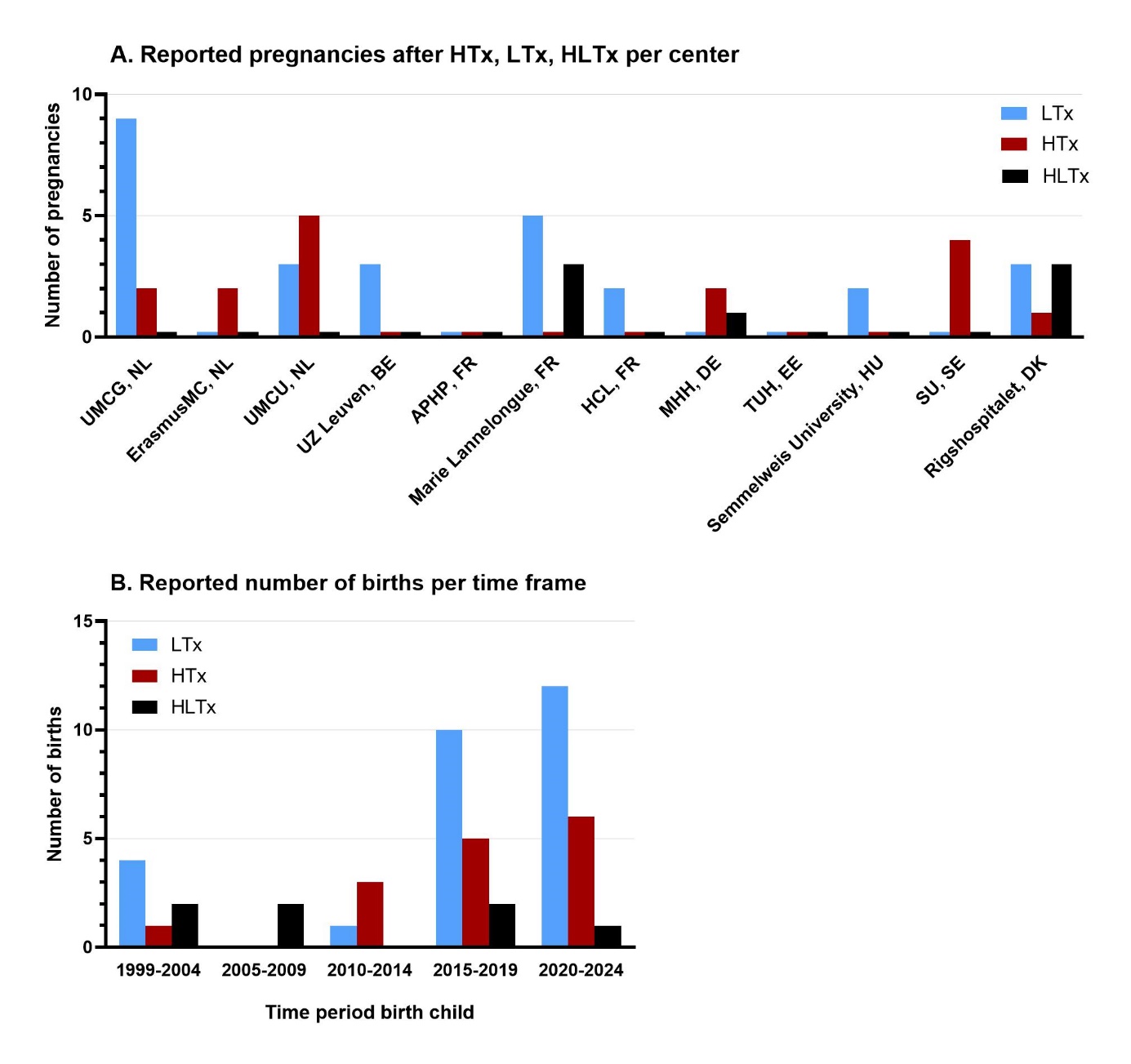


Number of pregnancies per center (A) and per timer period (B). *Abbreviations: APHP: Assistance publique, Hôpitaux de Paris, BE: Belgium, DE: Germany, DK: Denmark, EE: Estonia, ErasmusMC: Erasmus Medical Center, FR: France, HCL: Hospices Civils de Lyon, HLTx: heart lung transplantation, HU: Hungary, HTx: heart transplantation, LTx: lung transplantation, MHH: Medizinische Hochschule Hannover, NL: Netherlands, SE: Sweden, SU: Sahlgrenska University, TUH: Tartu University Hospital, UMCG: University Medical Center Groningen, UMCU: Univeristy Medical Center Utrecht, UZ Leuven: University Hospitals Leuven.*

*,*
